# Special Issue: Gut Microbiota in Disease and Health 2.0

**DOI:** 10.3390/ijms25084344

**Published:** 2024-04-14

**Authors:** Dulcenombre Gómez Garre, Javier Modrego

**Affiliations:** 1 Microbiota and Cardiovascular Risk Laboratory, Hospital Clínico San Carlos, Instituto de Investigación Sanitaria San Carlos (IdISSC), 28040 Madrid, Spain; 2 Network Biomedical Research Center for Cardiovascular Diseases (CIBERCV), Instituto de Salud Carlos III, 28029 Madrid, Spain; 3 Physiology Department, School of Medicine, Universidad Complutense, 28040 Madrid, Spain

In recent years, research on the gut microbiota has undeniably captivated the attention of students, investigators, clinicians, and the general public. The emergence of advanced next-generation sequencing platforms, the integration of multi-omics data, and the evolution of bioinformatics have revolutionized our understanding of gut microbial community composition, profoundly altering our comprehension of physiological and pathological processes.

The gut microbiota comprises a rich microbial community primarily dominated by bacteria but also encompassing populations of fungi, viruses, archaea, and protists. This intricate ecosystem appears to have evolved alongside humans, fostering a symbiotic relationship characterized by essential complementary metabolic functions not fully developed within the human host. 

One of the most remarkable aspects of the gut microbiota is its extraordinary adaptability and resilience. Throughout our lives, these microbial populations undergo dynamic fluctuations in response to several environmental factors, including diet, lifestyle, medications, or host genetics. These perturbations can significantly alter the composition and function of the gut microbiota, shaping its metabolic output and consequently modulating host physiology in profound ways. However, the precise roles of these microorganisms and their interactions with the host in health and disease remain unclear. 

Currently, there is growing interest in harnessing the therapeutic potential of the gut microbiota for the prevention and treatment of various diseases. Strategies aimed at modulating the gut microbiome, such as dietary interventions, probiotics, prebiotics, and faecal microbiota transplantation, hold promise for restoring microbial equilibrium and promoting host health.

Additionally, there has been a notable emergence of large-scale multi-geographic cohorts, marking significant progress beyond previous purely descriptive studies with limited study populations. In this regard, there are continual discoveries of new associations with a wide spectrum of human diseases. In response to this challenge, this Special Issue aims to offer a comprehensive update on the various aspects of recent gut microbiota research. Through seven papers, researchers addressed critical and miscellaneous gut-microbiota-related topics in order to emphasize its importance in promoting human health and preventing disease. 

The very first studies on gut microbiota focused on several intestinal pathologies, such as Crohn’s disease, irritable bowel syndrome, and ulcerative colitis, logically because these microbial populations live in that ecological niche of our body. However, in this Special Issue, we covered other diverse topics, such as the relationship between the gut microbiota and ophthalmology. Specifically, some lines of evidence suggest a potential link between gut dysbiosis and the development and exacerbation of non-infectious uveitis [1,2]. Some authors reviewed the most recent studies associating gut dysbiosis and uveitis, both in animal and human models, and discussed how anti-TNF treatment could restore gut microbiota composition and predict the response of each patient to this therapy. 

Another hot topic related to the gut microbiota is cardiovascular disease, as this is the main cause of death worldwide. Consequently, two manuscripts related to atherosclerosis disease and heart failure were also published. It was discussed how the gut microbiome, including metabolites of bacterial origin, is directly involved in all steps leading to atherogenesis, as well as in the main cardiovascular risk factors, such as hypertension, obesity, diabetes, and dyslipemia. In this line of evidence, the clinical improvement of patients who experienced their first episode of heart failure is associated with restored gut microbiota, mainly short-chain fatty acid bacteria genera producers. In this regard, butyrate is linked with improved inflammation levels and endothelial function during the one-year follow-up of these patients.

Bile acids undergo biotransformation with gut bacteria through processes such as deconjugation and dihydroxylation, influencing the composition and concentrations of bile acids, as well as their signalling properties [3]. Regarding cholestasis-induced liver disease, bile flow is impaired, resulting in the accumulation of bile acids in the liver and bloodstream. Moreover, bile acids serve as signalling molecules that regulate various metabolic processes through the activation of nuclear and membrane receptors, such as the FXR (farnesoid X receptor) and TGR5 (Takeda G protein-coupled receptor 5) [4]. These receptors are not only expressed in the liver but also in the intestine and other tissues, forming a bidirectional communication axis known as the gut–liver axis. In an excellent study, researchers found that bile conduct ligation and antibiotic-induced microbiome-depleted mice exhibit decreased gut microbiota abundance and diversity; as a consequence, mice develop a more accurate liver injury. In addition, they observed an inhibited liver detoxification enzyme expression, as well as elevated proinflammatory factors, leading to disturbed lipid metabolism.

The relationship between the gut microbiota and microscopic colitis, a type of inflammatory bowel disease characterized by chronic non-bloody diarrhoea and inflammation, is a condition with an unknown pathogenesis. Some pathophysiological explanations could be immune dysregulation, as well as bacterial translocation to bloodstream and microbial metabolites [5,6]. An exhaustive systematic review reveals that microbiome composition is potentially altered in microscopic colitis, with the main finding being a decreased *Akkermansia* population in faecal samples from these patients. However, no consistent findings regarding other taxonomic genera compared with healthy individuals were observed. 

Nutritional interventions also found a place in this Special Issue. In this sense, *Durvillaea antarctica*, also known as Antarctic seaweed or cochayuyo, is a large brown algae native to the Southern Hemisphere. This seaweed contains a variety of polysaccharides, fibres, and other bioactive compounds that may serve as prebiotics, promoting the growth and activity of beneficial bacteria in the gut. Different bioactive compounds found in *Durvillaea antarctica* may also possess immunomodulatory properties, influencing the activity and function of the immune system [7,8]. In a narrative review, investigators showed how *Durvillaea antarctica* could function as a prebiotic to modulate the gut microbiota and mediate anti-inflammatory, lipid-lowering, and hypoglycemic effects. The potential of this seaweed is based on certain polysaccharides that may modulate the concentrations of some deleterious metabolites (such as phenol or p-cresol) derived from some bacterial genera. Therefore, the authors emphasized that the performance of well-designed randomized clinical trials that provide a more comprehensive explanation of the potential health benefits of *Durvillaea antarctica* are needed.

A manuscript discussing COVID-19 and its relation with gut microbiota was also essential. Researchers provided thought-provoking findings highlighting the importance of maintaining good intestinal health in order to prevent viral infections, such as the one caused by SARS-CoV2 in the recent pandemic that struck our planet. The authors also suggested that future studies could focus on gut viromes and gut mycobiomes which play a role in COVID-19 [9,10]. 

To conclude, we invite our readers to embark on this enlightening journey through the captivating world of the gut microbiota. As we unravel its mysteries and decipher its role in human health and disease, we hope that this exploration inspires further research and innovations in the promotion of optimal human well-being.
